# High-Performance P- and N-Type SiGe/Si Strained Super-Lattice FinFET and CMOS Inverter: Comparison of Si and SiGe FinFET

**DOI:** 10.3390/nano13081310

**Published:** 2023-04-08

**Authors:** Yi-Ju Yao, Ching-Ru Yang, Ting-Yu Tseng, Heng-Jia Chang, Tsai-Jung Lin, Guang-Li Luo, Fu-Ju Hou, Yung-Chun Wu, Kuei-Shu Chang-Liao

**Affiliations:** 1 College of Semiconductor Research, National Tsing Hua University, Hsinchu 30013, Taiwan; yiju0410@gmail.com; 2 Department of Engineering and System Science, National Tsing Hua University, Hsinchu 30013, Taiwan; judy885502@gmail.com (C.-R.Y.); kcf300046@gmail.com (T.-Y.T.); aaa56246851@gmail.com (H.-J.C.); jung991005@gmail.com (T.-J.L.); 3 Taiwan Semiconductor Research Institute, Hsinchu 30078, Taiwan; glluo@narlabs.org.tw (G.-L.L.); hfg@narlabs.org.tw (F.-J.H.)

**Keywords:** super-lattice, strain, SiGe, FinFET, inverter, ON–OFF current ratio

## Abstract

This research presents the optimization and proposal of P- and N-type 3-stacked Si_0.8_Ge_0.2_/Si strained super-lattice FinFETs (SL FinFET) using Low-Pressure Chemical Vapor Deposition (LPCVD) epitaxy. Three device structures, Si FinFET, Si_0.8_Ge_0.2_ FinFET, and Si_0.8_Ge_0.2_/Si SL FinFET, were comprehensively compared with HfO_2_ = 4 nm/TiN = 80 nm. The strained effect was analyzed using Raman spectrum and X-ray diffraction reciprocal space mapping (RSM). The results show that Si_0.8_Ge_0.2_/Si SL FinFET exhibited the lowest average subthreshold slope (SS_avg_) of 88 mV/dec, the highest maximum transconductance (G_m, max_) of 375.2 μS/μm, and the highest ON–OFF current ratio (I_ON_/I_OFF_), approximately 10^6^ at V_OV_ = 0.5 V due to the strained effect. Furthermore, with the super-lattice FinFETs as complementary metal–oxide–semiconductor (CMOS) inverters, a maximum gain of 91 *v/v* was achieved by varying the supply voltage from 0.6 V to 1.2 V. The simulation of a Si_0.8_Ge_0.2_/Si super-lattice FinFET with the state of the art was also investigated. The proposed Si_0.8_Ge_0.2_/Si strained SL FinFET is fully compatible with the CMOS technology platform, showing promising flexibility for extending CMOS scaling.

## 1. Introduction

As CMOS technology scales down, Si-based FinFET technology will eventually reach its physical and electrical limits. To scale transistors, it is necessary to enhance the mobility of the channel. One approach to achieving this is through the use of high-mobility channel materials, such as SiGe [1,2], Ge [3,4], and GeSn [5,6], as well as strain engineering [7,8]. By applying mechanical stress to the channel region of the transistor, carrier mobility can be increased by modifying the band structure. Compressive strained SiGe is a promising alternative to Si in pFETs due to its superior hole mobility, which can enhance device drive current and transconductance (G_m_) [9,10,11]. On the other hand, tensile strained Si is proposed for enhancing effective electron mobility in nFETs [12,13,14]. Previous research has demonstrated that SiGe super-lattice-like heterostructures with a buried channel within bulk FinFETs can improve channel mobility and drive current, while also inhibiting short channel effects (SCEs) that are typically observed in conventional Si FinFET and SiGe FinFET devices [15]. The SiGe/Si super-lattice-like heterostructure induces an intrinsic strain effect on the high-mobility SiGe layer, resulting in even higher carrier mobility than traditional Si channels. Moreover, the heterostructure can be seamlessly integrated into existing FinFET technology, as it is compatible with current fabrication processes.

This paper presents the fabrication of a super-lattice channel FinFET using a Si_0.8_Ge_0.2_/Si heterostructure. The epitaxy mono-crystallinity and full strain of the Si_0.8_Ge_0.2_/Si super-lattice structure were confirmed through various material analyses. Conventional Si FinFET and Si_0.8_Ge_0.2_ FinFET were also prepared as references for device characteristic comparison. The results showed that the Si_0.8_Ge_0.2_/Si super-lattice FinFETs exhibit superior electrical characteristics in both N- and P-type devices. The potential use of the Si_0.8_Ge_0.2_/Si super-lattice FinFETs in CMOS inverters and the voltage transfer characteristics (VTCs) were also investigated. In addition, the Si_0.8_Ge_0.2_/Si super-lattice FinFET was simulated using the Sentaurus technology computer-aided design (TCAD) simulator with the state of the art. Overall, these results demonstrate that these devices are promising candidates for CMOS scaling.

## 2. Device Fabrication

**Figure 1:**
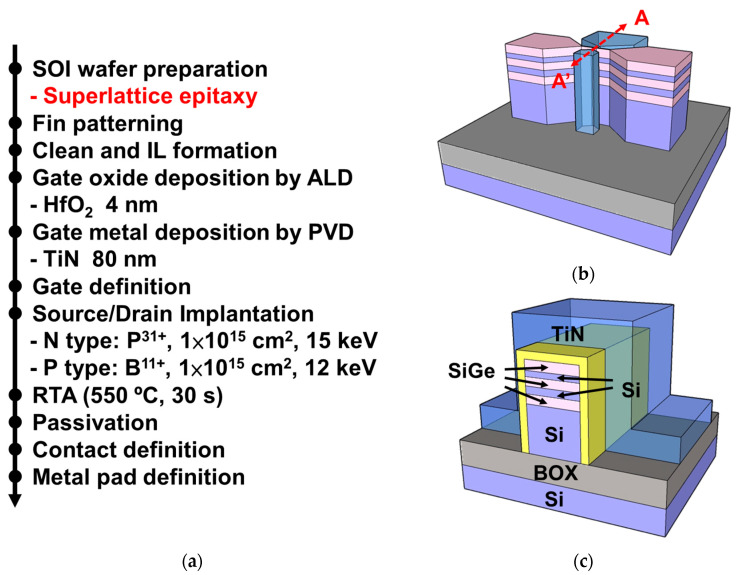
(**a**) Process flow for fabricating the Si_0.8_Ge_0.2_/Si SL FinFET. (**b**) Three-dimensional schematic and (**c**) A–A’ cross-sectional view of the Si_0.8_Ge_0.2_/Si SL FinFET.

Figure 1a depicts the fabrication process flow of the Si_0.8_Ge_0.2_/Si SL FinFET device. The structural and A–A’ cross-sectional schemes of the Si_0.8_Ge_0.2_/Si SL FinFET are illustrated in Figure 1b,c, respectively. First, the monocrystalline Si layer of an 8” SOI wafer was thinned down to the thickness of 25 nm, serving as the bottom layer. Then, a 5 nm thick Si_0.8_Ge_0.2_ layer was grown epitaxially, followed by two cycles of epitaxy consisting of a 5 nm thick Si layer/5 nm thick Si_0.8_Ge_0.2_ by LPCVD. The final total height of the Si_0.8_Ge_0.2_/Si SL stack was approximately 48 nm. For a fair comparison, conventional Si and Si_0.8_Ge_0.2_ FinFETs were also fabricated.

The active layer of the device was defined using e-beam lithography (EBL), and the pattern was transferred through reactive ion etching (RIE). After chemical cleaning, a 4 nm thick HfO_2_ layer was deposited as the gate dielectric layer using atomic layer deposition (ALD). Subsequently, the gate electrode was formed by depositing an 80 nm thick TiN metal using physical vapor deposition (PVD), and the pattern was transferred through EBL and RIE. For the N-type devices, the source/drain (S/D) regions were implanted with phosphorus at a dose of 1 × 10^15^ cm^−2^ and energy of 15 keV, and for P-type devices, the S/D regions were implanted with boron at a dose of 1 × 10^15^ cm^−2^ and energy of 12 keV. Rapid thermal annealing (RTA) was performed at 550 °C for 30 s in a nitrogen atmosphere to activate the dopants. Finally, the device fabrication was completed by performing oxide passivation, contact holes, and metallization processes.

## 3. Results and Discussion

**Figure 2:**
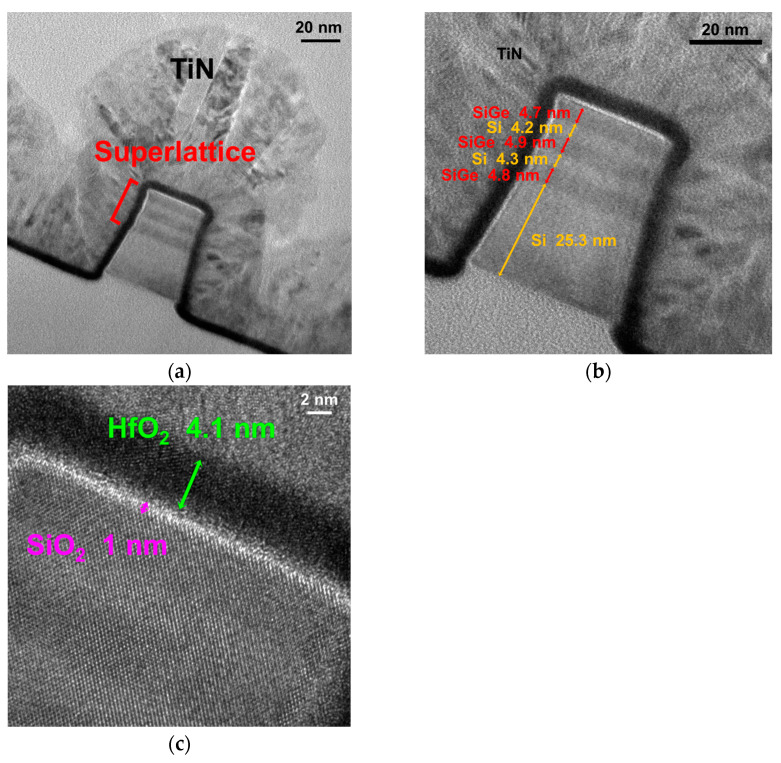
(**a**) TEM image of the Si_0.8_Ge_0.2_/Si SL FinFET with high uniform epitaxy, enlarged view of (**b**) the Si_0.8_Ge_0.2_/Si SL structure with each thickness, and (**c**) interfacial layer of SiO_2_ and HfO_2_.

**Figure 3:**
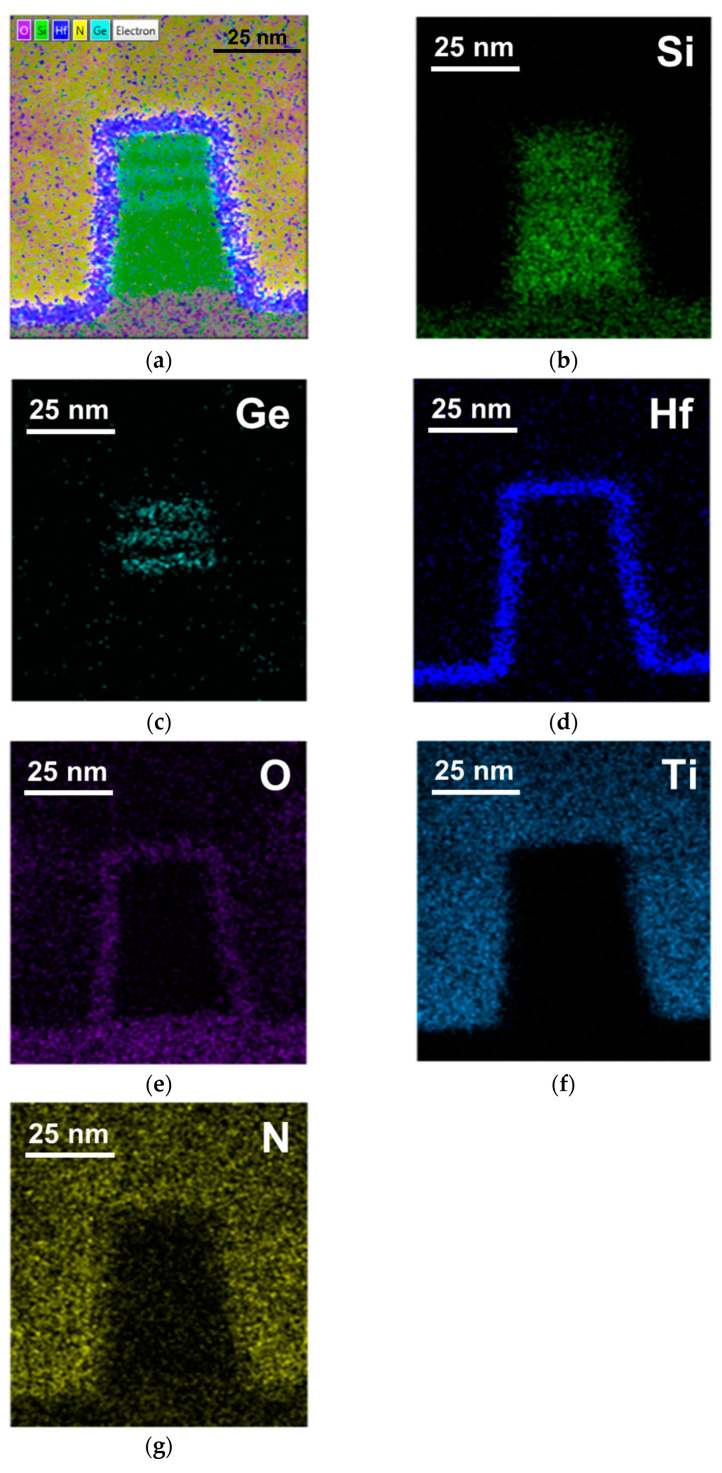
(**a**) EDX mapping of the Si_0.8_Ge_0.2_/Si SL FinFET and (**b**–**g**) the element distribution with clear separation between the Si_0.8_Ge_0.2_ and Si epitaxial layers.

Figure 2a depicts the transmission electron microscope (TEM) image of Si_0.8_Ge_0.2_/Si SL FinFET with high-quality epitaxy and an enlarged view of the Si_0.8_Ge_0.2_/Si SL structure. The thicknesses of each epitaxy layer are shown in Figure 2b, where the a three-stacked epi-layer SL structure has a total fin height (F_H_) of 48 nm. Figure 2c displays an enlarged view of the 1 nm thick SiO_2_ interfacial layer and 4.1 nm thick HfO_2_ gate insulator. The corresponding energy-dispersive X-ray spectroscopy (EDX) mapping is presented in Figure 3a. Clear separation of the Si_0.8_Ge_0.2_ and Si epi-layers can be observed in the EDX mapping in Figure 3b–g, which also show the distributions of the elements including Si, Ge, Hf, O, Ti, and N.

**Figure 4:**
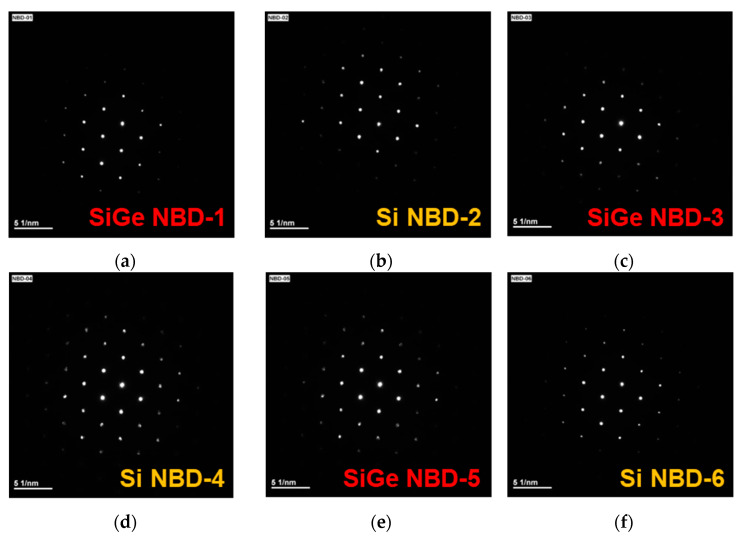
(**a**–**f**) NBD patterns of Si_0.8_Ge_0.2_/Si SL FinFET from top to bottom layer reveals well epitaxial and single-crystal structure.

**Figure 5:**
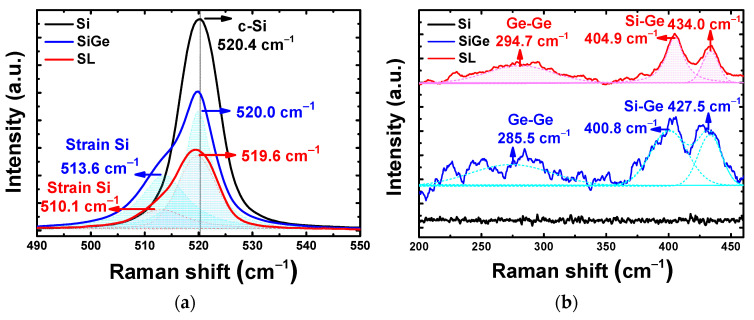
Raman spectra of Si, Si_0.8_Ge_0.2_, and Si_0.8_Ge_0.2_/Si SL samples of (**a**) the Si substrate and strain Si components and (**b**) Si-Ge and Ge-Ge vibration mode.

To verify the crystallinity of the Si_0.8_Ge_0.2_/Si SL heterostructure, nanobeam diffraction (NBD) analysis was performed, with a focus on each layer. The NBD patterns according to the focusing point from the top to the bottom layer of the Si_0.8_Ge_0.2_/Si SL FinFET are presented in Figure 4a–f. The sharp diffraction patterns reveal that the six Si_0.8_Ge_0.2_/Si SL layers are single-crystalline and epitaxially grown. Figure 5 shows the Raman spectra of Si, Si_0.8_Ge_0.2_, and Si_0.8_Ge_0.2_/Si SL samples to analyze the strain effect. Both Si_0.8_Ge_0.2_ and Si_0.8_Ge_0.2_/Si SL samples exhibit the Si-Si vibration mode, attributed to tensile strained Si, with lower wavenumbers than the c-Si peak (Figure 5a) [16], with the Si_0.8_Ge_0.2_/Si SL sample exhibiting more tensile strain. Furthermore, the Ge-Ge and Si-Ge vibration modes of the Si_0.8_Ge_0.2_/Si SL samples were observed to shift to higher wavenumbers compared to the Si_0.8_Ge_0.2_ sample, indicating that the Si_0.8_Ge_0.2_ layers in the Si_0.8_Ge_0.2_/Si SL sample are more compressively strained (Figure 5b).

**Figure 6:**
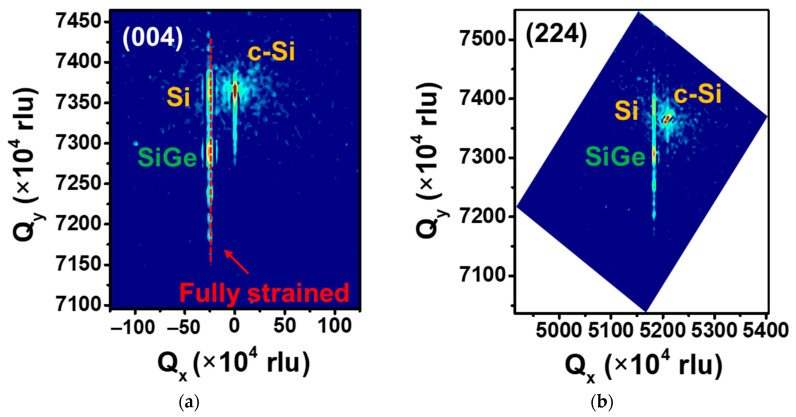
XRD RSM of Si_0.8_Ge_0.2_/Si SL sample in the direction (**a**) (004) and (**b**) (224), showing they are fully strained.

XRD reciprocal space mapping (RSM) was used to analyze the Si_0.8_Ge_0.2_/Si SL sample at the (004) and (224) planes. The X-ray reflection from Si_0.8_Ge_0.2_/Si and Si appeared at the same in-plane wave vector, indicating that they have the same in-plane lattice constant (Figure 6a,b) [17]. This indicated that the Si_0.8_Ge_0.2_ in the Si_0.8_Ge_0.2_/Si SL sample is fully compressively strained since the lattice constant of single crystal Si and Ge are 5.43 Å and 5.66 Å, respectively. The mole fraction of Ge in SiGe layers, which is approximately 20%, was determined by XRD through Vegard’s law [18].

**Figure 7:**
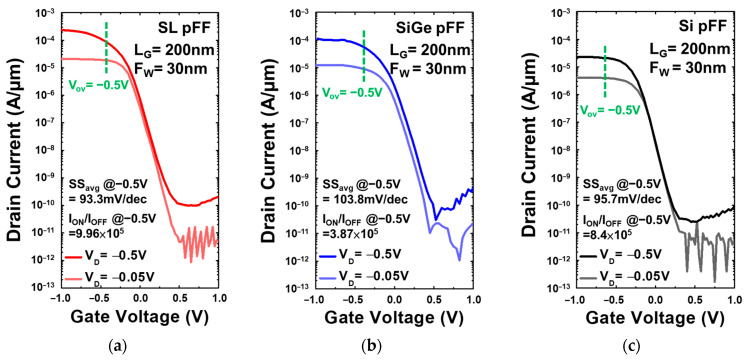
I_D_-V_G_ characteristics of P-type (**a**) Si_0.8_Ge_0.2_/Si SL FinFET, (**b**) Si_0.8_Ge_0.2_ FinFET, and (**c**) Si FinFET. The I_D_ is normalized by the footprint width, and V_TH_ is extracted at a constant I_D_ of 10^−7^ A/µm. I_ON_ is extracted at V_OV_ = V_D_ = −0.5 V.

**Figure 8:**
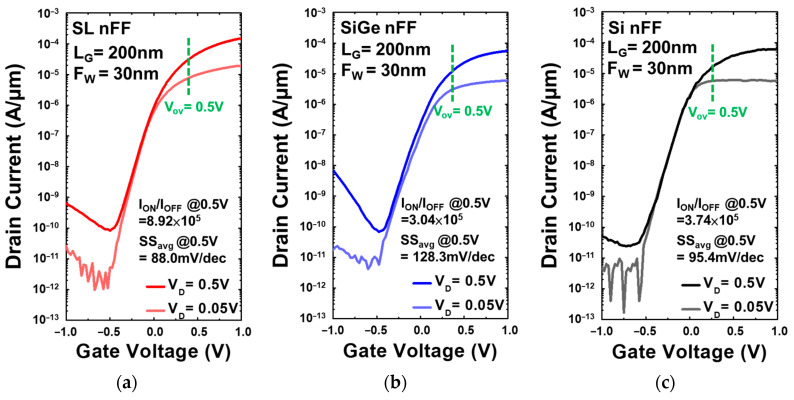
I_D_-V_G_ characteristics of N-type (**a**) Si_0.8_Ge_0.2_/Si SL FinFET, (**b**) Si_0.8_Ge_0.2_ FinFET, and (**c**) Si FinFET. The I_D_ is normalized by the footprint width, and V_TH_ is extracted at a constant I_D_ of 10^−7^ A/µm. I_ON_ is extracted at V_OV_ = V_D_ = 0.5 V.

Figure 7 and Figure 8 compare the characteristics of drain current (I_D_) versus gate voltage (V_G_) curves for Si_0.8_Ge_0.2_/Si SL, Si_0.8_Ge_0.2_, and Si FinFET devices with HfO_2_ = 4 nm/TiN = 80 nm, Fin width (F_w_) = 30 nm, and gate length (L_G_) = 200 nm for both P-type (Figure 7a–c) and N-type (Figure 8a–c). The Si_0.8_Ge_0.2_/Si SL FinFETs exhibit the lowest SS_avg,P_ = 93.3 mV/dec, SS_avg,N_ = 88.0 mV/dec atc, where SS was extracted from every adjacent measured point. Due to the strain effect of the super-lattice structure, the Si_0.8_Ge_0.2_/Si SL FinFET has the highest I_ON_ and reflects on the high I_ON_/I_OFF_ of approximately 10^6^. The I_D_ is normalized by the footprint width, and V_TH_ is extracted at a constant I_D_ of 10^−7^ A/µm. I_ON_ is extracted at V_OV_ = V_D_ = ±0.5 V. All electrical characteristics were determined using Keithley 4200A at room temperature.

**Figure 9:**
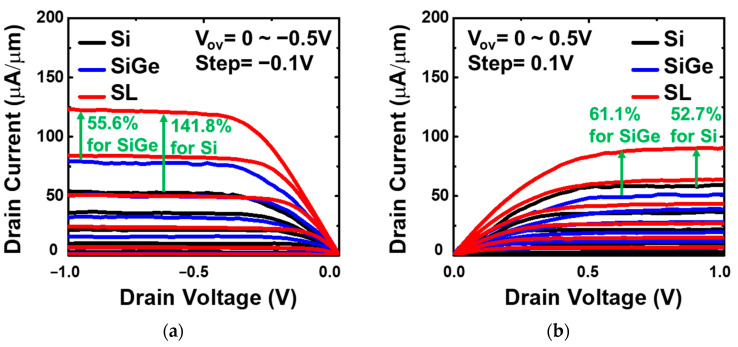
I_D_-V_D_ characteristics of (**a**) P-type Si_0.8_Ge_0.2_/Si SL, Si_0.8_Ge_0.2_, and Si FinFET and (**b**) N-type Si_0.8_Ge_0.2_/Si SL, Si_0.8_Ge_0.2_, and Si FinFET with L_G_ = 200 nm and F_W_ = 30 nm.

**Figure 10:**
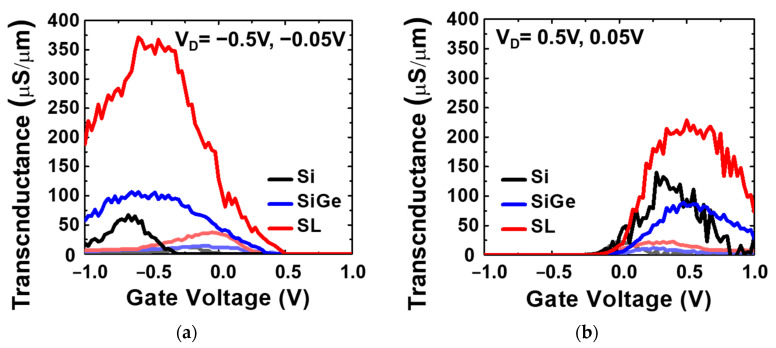
G_m_ characteristics of (**a**) P-type Si_0.8_Ge_0.2_/Si SL, Si_0.8_Ge_0.2_, and Si FinFET and (**b**) N-type Si_0.8_Ge_0.2_/Si SL, Si_0.8_Ge_0.2_, and Si FinFET with L_G_ = 200 nm and F_W_ = 30 nm.

Figure 9a shows that the Si_0.8_Ge_0.2_/Si SL FinFET has a drive current (V_OV_ = V_D_ = ±0.5 V) of 124.6 µA/µm, which is 141.8% and 55.6% higher than that of the Si FinFET and Si_0.8_Ge_0.2_ FinFET, respectively, for the P-type. In Figure 9b, the drive current for the Si_0.8_Ge_0.2_/Si SL FinFET is 92.5 µA/µm, which is 52.7% and 61.1% higher than that of the Si FinFET and Si_0.8_Ge_0.2_ FinFET, respectively, for the N-type. Figure 10a,b show that the Si_0.8_Ge_0.2_ SL FinFETs exhibit the highest maximum transconductance (G_m,P_) of 375.2 µS/µm and G_m,N_ 235.1 µS/µm at V_D_ = ±0.5V.

**Figure 11:**
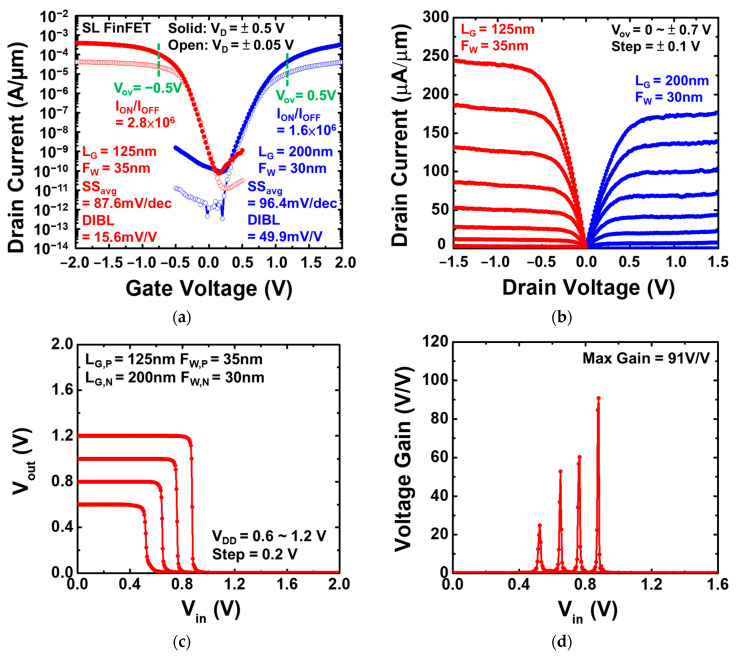
(**a**) I_D_-V_G_ and (**b**) I_D_-V_D_ characteristics of the Si_0.8_Ge_0.2_/Si SL FinFET CMOS inverter. (**c**) VTC and (**d**) gain of the Si_0.8_Ge_0.2_/Si SL FinFET CMOS inverters with the V_DD_ ranging from 0.6 to 1.2 V.

Figure 11a displays the I_D_-V_G_ characteristics of the CMOS inverter operation using the Si_0.8_Ge_0.2_/Si SL FinFETs, with F_W_ = 35 nm and L_G_ = 125 nm for the P-type and F_W_ = 30 nm and L_G_ = 200 nm for the N-type. The Si_0.8_Ge_0.2_/Si SL FinFET CMOS inverter exhibits SS_avg,P_ = 87.6 mV/dec, SS_avg,N_ = 96.4 mV/dec, the drain-induced barrier lowering (DIBL) values of DIBL_P_ = 15.6 mV/V, DIBL_N_ = 49.9 mV/V, and high I_ON_/I_OFF_ greater than 10^6^ at V_D_ = ±0.5V. In Figure 11b, the drive current (V_OV_ = V_D_ = ±0.7 V) of the Si_0.8_Ge_0.2_/Si SL FinFET CMOS inverter is 245.9 µA/µm and 176.8 µA/µm for the P- and N-type Si_0.8_Ge_0.2_/Si SL FinFET, respectively. Figure 11c depicts the voltage transfer characteristic (VTC) of the Si_0.8_Ge_0.2_/Si SL FinFET CMOS inverter, with a maximum gain of 91 *v*/*v* by varying the supply voltage from 0.6 V to 1.2 V, as shown in Figure 11d.

**Figure 12:**
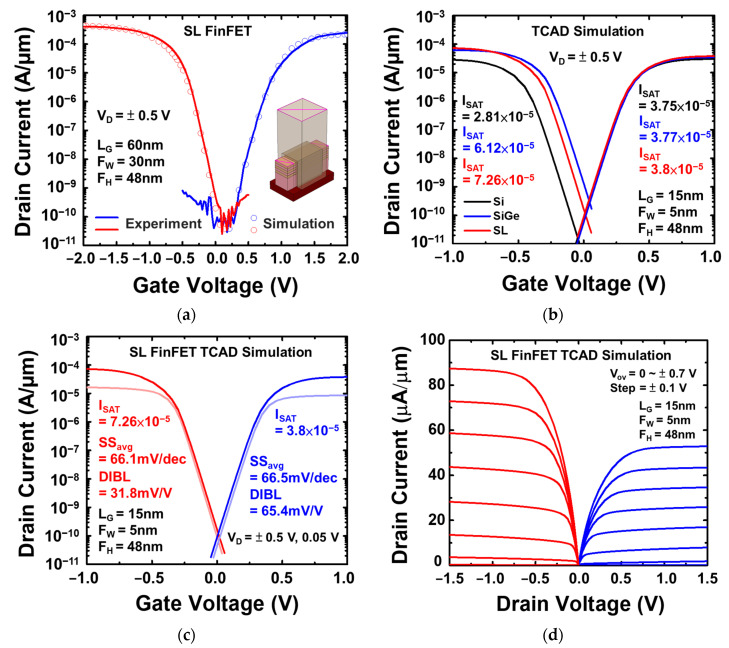
(**a**) The calibrated I_D_-V_G_ characteristic of the Si_0.8_Ge_0.2_/Si SL FinFET between experimental data and TCAD simulation. (**b**) The simulated I_D_-V_G_ characteristics of Si_0.8_Ge_0.2_/Si SL, Si_0.8_Ge_0.2_, and Si FinFET for comparison, (**c**) the simulated I_D_-V_G_ and (**d**) I_D_-V_D_ characteristics of Si_0.8_Ge_0.2_/Si SL FinFET, both with L_G_ = 15 nm and F_W_ = 5 nm according to state of the art and a total fin height of 48 nm and HfO_2_ = 4nm, as used in the experiment of this work.

To confirm the potential of the proposed approach in contributing to the development of future technology nodes, the Sentaurus TCAD simulator was applied for simulation. The simulation parameters of the devices, including L_G_ = 15 nm and F_W_ = 5 nm, were selected based on the current state of the art [19]. The gate insulator utilized was HfO_2_ with a thickness of 4 nm, and the total fin height was set to 48 nm to match the experimental conditions of this study. Figure 12a displays the calibrated I_D_-V_G_ characteristic of the Si_0.8_Ge_0.2_/Si SL FinFET with L_G_ = 60 nm and F_W_ = 30 nm for both the P- and N-type, between experimental data and the TCAD simulation results. In Figure 12b, the I_D_-V_G_ characteristics of the Si_0.8_Ge_0.2_/Si SL, Si_0.8_Ge_0.2_, and Si FinFET devices are compared. The results indicate that compared to the Si FinFET and Si_0.8_Ge_0.2_ FinFET, the saturation current of the Si_0.8_Ge_0.2_/Si SL FinFET exhibits the highest value of 7.26 × 10^−5^ for the P-type and 3.8 × 10^−5^ for the N-type. Figure 12c presents the simulated Si_0.8_Ge_0.2_/Si SL FinFET, which exhibits SS_avg,P_ = 66.1 mV/dec and SS_avg,N_ = 66.5 mV/dec, with values of DIBL_P_ = 31.8 mV/V and DIBL_N_ = 65.4 mV/V. In Figure 12d, the drive current (V_OV_ = V_D_ = ±0.7 V) of the Si_0.8_Ge_0.2_/Si SL FinFET is 87.4 µA/µm for the P-type and 52.8 µA/µm for the N-type. While GAAFET has the advantage of better scaling, its complex fabrication and low yield make it less practical for certain applications. As an alternative, the Si_0.8_Ge_0.2_/Si SL FinFET can extend FinFET technology to more particle applications.

## 4. Conclusions

The Si_0.8_Ge_0.2_/Si SL FinFET and CMOS inverter were both fabricated and characterized to evaluate their high-mobility channel achieved through the strain effect. The crystallinity and strain effect were confirmed through the implementation of NBD, Raman scattering, and XRD RSM. Si_0.8_Ge_0.2_/Si SL FinFETs exhibit remarkable electrical characteristics, including SS_avg_ = 88 mV/dec, G_m, max_ = 375.2 μS/μm, and the highest I_ON_/I_OFF_, approximately 10^6^, when compared to conventional Si and Si_0.8_Ge_0.2_ FinFETs. The Si_0.8_Ge_0.2_/Si SL FinFET CMOS inverter exhibits SS_avg,P_ = 87.6 mV/dec, SS_avg,N_ = 96.4 mV/dec, the DIBL values of DIBL_P_ = 15.6 mV/V and DIBL_N_ = 49.9 mV/V, and high I_ON_/I_OFF_ greater than 10^6^ at V_D_ = ±0.5V, with a maximum gain of 91 *v/v* by varying the supply voltage from 0.6 V to 1.2 V. The simulation of the Si_0.8_Ge_0.2_/Si SL FinFET with the state of the art demonstrates the potential for extending FinFET technology. The purposed Si_0.8_Ge_0.2_/Si strained SL FinFET is fully compatible with the CMOS technology platform, making it a promising alternative for extending future nanoelectronics applications.

## Data Availability

The data presented in this study are available on request from the corresponding author.
